# Niclosamide reduces glucagon sensitivity via hepatic PKA inhibition in obese mice: Implications for glucose metabolism improvements in type 2 diabetes

**DOI:** 10.1038/srep40159

**Published:** 2017-01-05

**Authors:** Md. Kamrul Hasan Chowdhury, Nigel Turner, Nicholas L. Bentley, Abhirup Das, Lindsay E. Wu, Dulama Richani, Sonia Bustamante, Robert B. Gilchrist, Margaret J. Morris, Peter R. Shepherd, Greg C. Smith

**Affiliations:** 1 Depatment of Pharmacology, School of Medical Sciences, UNSW Australia, Sydney, Australia; 2 School of Women’s & Children’s Health, UNSW Australia, Sydney, Australia; 3 Bioanalytical Mass Spectrometry Facility, UNSW Australia, Sydney, Australia; 4 Department of Molecular Medicine & Pathology, University of Auckland, Auckland, New Zealand

## Abstract

Type 2 diabetes (T2D) is a global pandemic. Currently, the drugs used to treat T2D improve hyperglycemic symptom of the disease but the underlying mechanism causing the high blood glucose levels have not been fully resolved. Recently published data showed that salt form of niclosamide improved glucose metabolism in high fat fed mice via mitochondrial uncoupling. However, based on our previous work we hypothesised that niclosamide might also improve glucose metabolism via inhibition of the glucagon signalling in liver *in vivo*. In this study, mice were fed either a chow or high fat diet containing two different formulations of niclosamide (niclosamide ethanolamine salt - NENS or niclosamide - Nic) for 10 weeks. We identified both forms of niclosamide significantly improved whole body glucose metabolism without altering total body weight or body composition, energy expenditure or insulin secretion or sensitivity. Our study provides evidence that inhibition of the glucagon signalling pathway contributes to the beneficial effects of niclosamide (NENS or Nic) on whole body glucose metabolism. In conclusion, our results suggest that the niclosamide could be a useful adjunctive therapeutic strategy to treat T2D, as hepatic glucose output is elevated in people with T2D and current drugs do not redress this adequately.

Type 2 diabetes (T2D) is a complex metabolic disorder characterised by hyperglycaemia which has now reached epidemic levels. It has been predicted that the current prevalence of 400 million people worldwide will double over the next 20 years^1^,^2^. T2D also doubles the risk of a wide range of cardiovascular diseases including coronary heart disease and stroke^3^, but also non-vascular diseases such as cancer, mental and nervous system disorders, infections and liver disease^4^. Current pathophysiological evidence describes T2D as a spectrum disorder characterised by variable degrees of insulin resistance and β-cell dysfunction^5^ which leads to hyperglycaemia. But despite many years of research into insulin signalling and T2D no fully effective solution to treat this metabolic disorder has been discovered.

There is strong evidence that glucagon signalling plays a vital role in maintaining hyperglycemia in people with T2D^6^,^7^,^8^. Glucagon activates its receptor to increase hepatic glucose production and output during fasting, a process that is essential in controlling our metabolic state under normal physiological conditions^9^. But under pathophysiological conditions such as T2D, hyperglucagonemia is strongly linked to maintaining high blood glucose levels^6^,^7^,^8^,^10^. It is well established that glucagon concentrations are elevated in both animal models of diabetes and people with T2D^11^,^12^,^13^. Long term hyperglucagonemia has been linked to increased hepatic glucose output leading to hyperglycaemia in T2D^14^. Under normal physiological conditions, glucagon decreases after a meal but in T2D it is either not reduced or in some cases increased further^6^,^7^,^8^. Likewise, suppression of postprandial hyperglucagonemia improves hyperglycemia in people with T2D^15^. Moreover, a mechanism leading to hyperglucagonemia in T2D has been proposed via reduced expression levels of the sodium-glucose transporter SGLT2 in glucagon secreting α-cells in T2D, thus leading to increased glucagon secretion^16^.

Recently, we identified novel glucagon responses in isolated rat liver showing that glucagon rapidly increases hepatic glucose production (HGP) and phosphorylates serine 552 on β-catenin, thereby increasing targeted β-catenin/TCF7L2 gene expression^17^. The effect was reversed by niclosamide, a drug used to treat intestinal infections of tapeworms in humans by uncoupling the mitochondria, by blocking glucagon induced PKA activation via cAMP signalling^18^. Recently, Tao *et al*.^19^ reported that the niclosamide ethanolamine salt showed efficacy in the treatment of diet induced diabetes in mice. They found that oral niclosamide ethanolamine salt treatment increased energy expenditure and lipid metabolism and reduced hepatic steatosis, which led to improved metabolism. They also found that oral niclosamide ethanolamine salt treatment in *db/db* mice improved glycaemic control and delayed disease progression^19^. However, it remains unclear if the FDA approved niclosamide shows similar efficacy to the niclosamide ethanolamine salt form of the drug.

The current study investigates the molecular effects of niclosamide on glucose metabolism *in vivo* in normal mice and mice with impaired glucose metabolism following high fat diet feeding. These studies demonstrate that niclosamide causes large improvements in glucose metabolism without affecting body composition or insulin sensitivity but instead via attenuation of glucagon signalling and hepatic glucose production.

## Results

### Comparing NENS and Nic mitochondrial uncoupling potential

**Figure 1:**
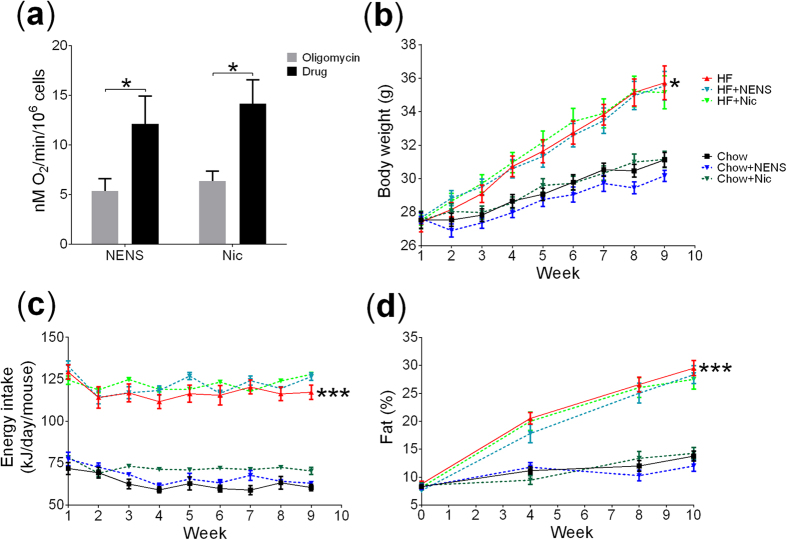
NENS or Nic uncouples mitochondrial respiration but has no effect on body weight, energy intake or body composition. (**a**) Oxygen consumption of isolated C57BL/6 hepatocytes using the Clark electrode with oligomycin and either NENS or Nic, (**b**) weekly body weight, (**c**) weekly food intake and (**d**) total fat % measurements using EcoMRI, as indicated in the time points. n = 12 per group. Statistical significance (P) was determined by multiple comparisons and repeated measures Two-way ANOVA test. The data is showed as the mean ± S.E.M. *p < 0.05 or ***p < 0.001 compared to the control chow or control high fat mice.

To confirm comparable drug efficacy between NENS and the FDA approved Nic we first undertook a study to examine their effects on mitochondrial uncoupling by measuring oxygen consumption in the presence of the F_0_F_1_ ATP synthase inhibitor oligomycin. Both NENS and Nic at concentration of 1 μM increased oxygen consumption in primary C57BL/6 mice hepatocytes and we found no difference in the uncoupling potential between these two drugs (Fig. 1a). A dose effect was also observed from 500 nM–5 μM (Supplementary Fig. S1).

### The effect of NENS or Nic on body composition and energy metabolism in mice

Because we found no difference in the uncoupling potential between NENS or Nic we used the same dose of each drug in our animal studies. Both NENS and Nic were dosed at 1,500 p.p.m. in high fat diet and at 750 p.p.m. in chow. This results in an approximate dose of 150 mg/kg body weight per 24 h period that has previously been effective in improving glucose metabolism^19^.

As expected, control animals fed the high fat diet significantly increased total body weight, compared to control chow fed mice (Fig. 1b). Interestingly, despite their effect to uncouple mitochondria both forms of niclosamide (NENS or Nic) had no impact on total body weight in mice consuming either the chow or high fat diet (Fig. 1b). As expected, high fat fed mice consumed significantly more calories per day, compared to chow fed mice but within each dietary group, no difference in calorie intake between the controls or drug exposed mice was found (Fig. 1c). Likewise, total body fat percentage was measured throughout the experiment and all groups on the high fat diet had significant increases in fat percentages compared to the chow fed groups. However, no difference between the controls or drug exposed groups was observed (Fig. 1d).

**Figure 2:**
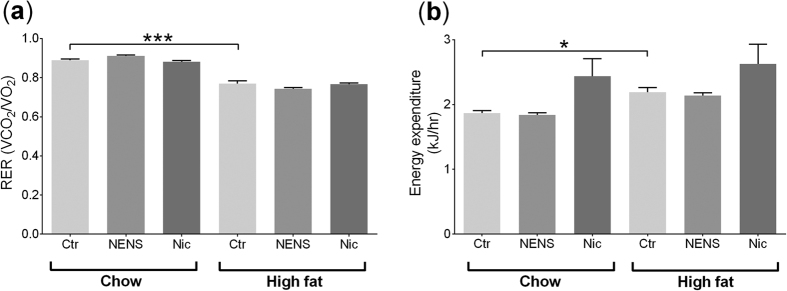
Ten weeks of NENS or Nic has no effect on energy expenditure (CLAMS) on chow or high fat diets. (**a**) The respiratory exchange ratio (RER) and (**b**) energy expenditure. n = 12 per group. Statistical significance (P) was determined by multiple comparisons Two-way ANOVA test. The data is showed as the mean ± S.E.M. *p < 0.05 or ***p < 0.001 compared to the control (Ctr) chow or control (Ctr) high fat mice.

We investigated the metabolic effect of oral NENS and Nic on mice consuming either the chow or high fat diets using indirect calorimetry. Animals maintained on the high fat diets had a lower respiratory exchange ratio (Fig. 2a) and higher energy expenditure (Fig. 2b) compared to the chow fed mice, but within each dietary group no differences were found with either NENS or Nic treatment. This indicates that animals fed the high fat diet used a larger proportion of energy expenditure from lipid oxidation but NENS or Nic did not increase this further.

### The effect of NENS or Nic on glucose metabolism in obese mice

**Figure 3:**
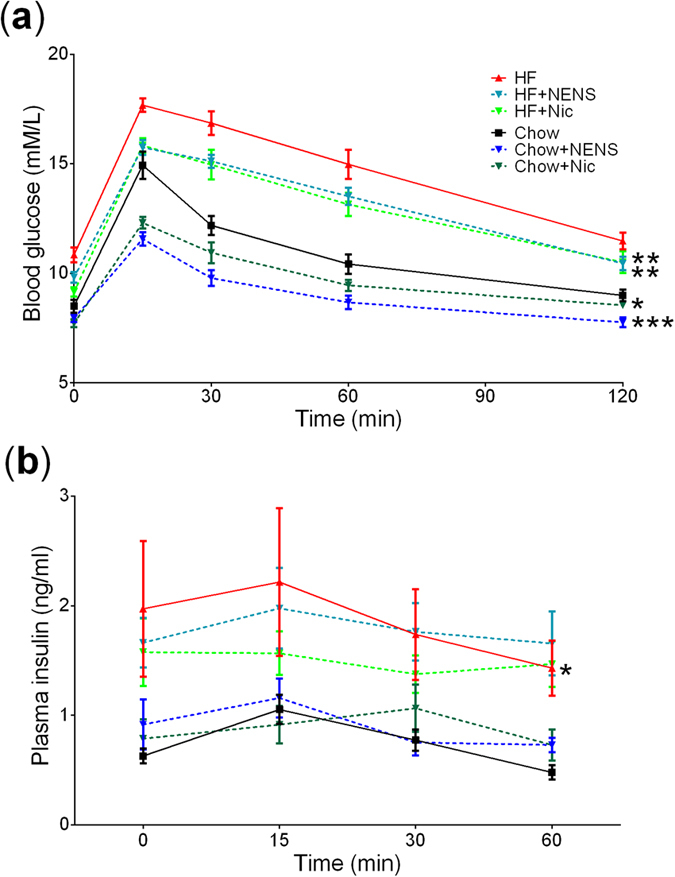
Both NENS and Nic improve glucose metabolism by reducing glucagon mediated hepatic glucose release. (**a**) Glucose tolerance test, fasted mice (8 am–2 pm) were injected with glucose solution i.p. and blood glucose measured at the indicated time points. (**b**) Insulin levels measurement from blood plasma. n = 12 per group. Statistical significance (P) was determined by repeated measures and multiple comparisons Two-way ANOVA test. The data is showed as the mean ± S.E.M. *p < 0.05, **p < 0.01 or ***p < 0.001 compared to the control (Ctr) chow or control (Ctr) high fat mice.

We undertook a number of metabolic tests to see if these drugs would improve glucose metabolism independent of body weight or energy expenditure. Using a glucose tolerance test (GTT), control mice on the high fat diet developed impaired glucose metabolism, compared to control mice on the chow diet (Fig. 3a). Interestingly, NENS and Nic significantly improved glucose metabolism across both diets, compared to control mice (Fig. 3a). To determine if the improvements in glucose metabolism are due to increased insulin secretion we quantified insulin levels at the time points 0, 15, 30 and 60 min during the GTT. As expected, control mice on a high fat diet had significantly higher plasma insulin levels compared to control mice on the chow diet (Fig. 3b). But we found no difference between the NENS or Nic groups within the dietary groups (Fig. 3b).

**Figure 4:**
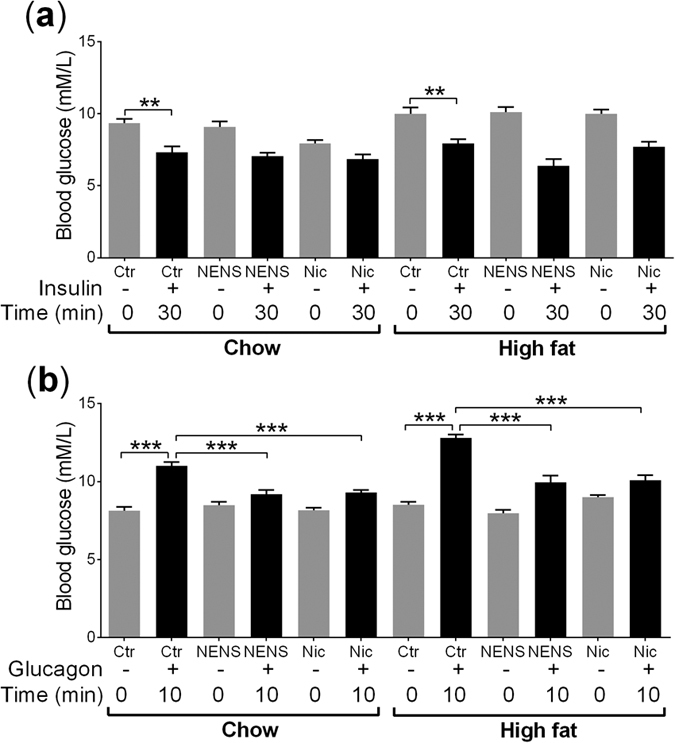
Both NENS and Nic improve glucose metabolism. (**a**) Insulin sensitivity test, fasted mice (8 am–2 pm) were injected with insulin i.p. and blood glucose measured at time 0 and 30 minutes post insulin exposure. (**b**) Glucagon sensitivity test, fasted mice (8 am–2 pm) were injected with glucagon i.p. and blood glucose measured at time 0 and 10 minutes post glucagon exposure. n = 12 per group. Statistical significance (P) was determined by multiple comparisons Two-way ANOVA test. The data is showed as the mean ± S.E.M. **p < 0.01 or ***p < 0.001 compared to the control (Ctr) chow or control (Ctr) high fat mice.

To see if the improvements in glucose metabolism, following NENS or Nic treatment are linked to increased sensitivity to insulin, we undertook an insulin sensitivity test. As expected, 30 minutes following insulin exposure blood glucose levels were significantly reduced in control and drug exposed mice on either the chow or high fat diets (Fig. 4a). However, no effect of NENS or Nic was observed (Fig. 4a). Therefore, neither NENS nor Nic impacted insulin sensitively that would explain the improvements in glucose metabolism.

We have previously found that Nic can inhibit the glucagon signalling pathway in primary hepatocytes^18^. Thus we undertook a glucagon sensitivity test in mice exposed to NENS or Nic to see if these drugs could block this pathway *in vivo.* As expected, 10 minutes following glucagon exposure control untreated mice on the chow or high fat diets showed a rapid and significant increase in circulating blood glucose levels (Fig. 4b). But in mice exposed to either NENS or Nic and maintained on the chow or high fat diets there was an attenuation in the glucagon induced rise in blood glucose levels consistent with the drug reducing hepatic glucose output (Fig. 4b). Thus, the improvement in glucose metabolism following chronic NENS or Nic exposure was potentially due to inhibition of the glucagon signalling pathway.

### The effect of NENS or Nic on the glucagon signalling pathway

**Figure 5:**
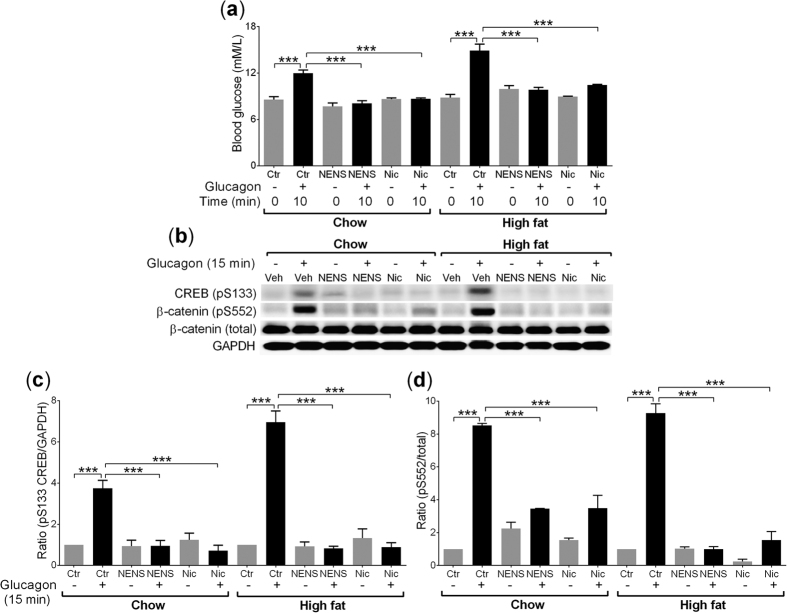
Both NENS and Nic block glucagon induced Ser133 (CREB) and Ser552 (β-catenin) phosphorylation on chow or high fat diets, leading to reduced hepatic glucose output, a downstream marker of PKA inhibition. (**a**) Blood glucose levels following glucagon exposure, non-fasted mice (8 am) were injected with glucagon i.p. and blood glucose measured at time 0 and 10 minutes, before rapid tissue collection. (**b**) A representative western blot image after 15 minutes of glucagon (1.15 nM) exposure on Ser133 (CREB) and Ser552 (β-catenin) phosphorylation from the liver. (**c**) Quantification of western blots for pSer133/GAPDH (CREB) and (**d**) pSer552/total (β-catenin). n = 6 per group. Statistical significance (P) was determined by multiple comparisons Two-way ANOVA test. The data is showed as the mean ± S.E.M. ***p < 0.001 compared to the control (Ctr) chow or control (Ctr) high fat mice.

Both NENS and Nic uncouple mitochondria, which would lead to reduced ATP formation and based on our *in vitro* data, would block PKA signalling which is an essential component of the glucagon signalling pathway^17^,^18^. Therefore, we tested the effect of NENS or Nic on glucagon responses in a terminal study in mice exposed to vehicle or glucagon for 15 minutes before cervical dislocation and rapid liver collection. As expected, after glucagon exposure control mice on either a chow or high fat diet showed significant increases in circulating glucose levels, compared to the 0 time point (Fig. 5a). As shown previously (Fig. 4b) both NENS and Nic exposed mice on either the chow or high fat diets had a significant reduction in glucagon induced increases in blood glucose after 10 minutes exposure, compared to the vehicle mice 10 minutes after glucagon (Fig. 5a).

To provide support that NENS and Nic block the glucagon signalling pathway, we used CREB serine 133 as a readout, because this is a widely used measure of PKA activity^20^,^21^,^22^. Control mice exposed to glucagon for 15 minutes on both the chow and high fat diets had a significant increase in serine 133 phosphorylation on CREB but both NENS and Nic blocked this effect (Fig. 5b,c). Thus, we have evidence that NENS and Nic block the glucagon signalling pathway that may underlie the improvement in glucose metabolism found with these drugs independent of body composition, energy expenditure, or insulin signalling.

We recently identified that glucagon leads to rapid phosphorylation of β-catenin via the glucagon signalling PKA pathway^18^. Therefore, we used serine 552 phosphorylation on β-catenin as another readout of PKA activity in liver samples from the mice exposed to NENS or Nic and glucagon. As expected, glucagon exposure for 15 minutes before tissue collection resulted in rapid phosphorylation of serine 552 on β-catenin in both chow and high fat fed vehicle control mice (Fig. 5b,d). Interestingly, 10 weeks of NENS or Nic exposure resulted in a significant inhibition of the glucagon signalling pathway on either the chow or the high fat diets (Fig. 5b). Therefore, NENS and Nic blocked the PKA signalling pathway *in vivo.*

## Discussion

We found that two different formulations of niclosamide cause significant improvements in glucose metabolism in both control and obese mice but this effect was not due to alterations in insulin sensitivity (although the gold standard insulin CLAMP may be required in future research to confirm this), or increased insulin secretion, or energy expenditure. Instead, we found that both NENS and Nic blocked hepatic glucagon signalling and glucagon induced increases in blood glucose levels. This would indicate that the liver is a major target of niclosamide’s effect on whole body glucose metabolism. Our results further indicate that these effects arise via a niclosamide induced suppression of PKA as indicated by reduced phosphorylation of CREB and β-catenin. PKA is well known to have an essential role in glucagon mediated hepatic glucose release from the liver^23^ and we have previously found that niclosamide reduces cAMP production following glucagon exposure and prevents PKA mediated phosphorylation of serine 552 on β-catenin using primary rat hepatocytes^18^.

As described earlier, the secretion of glucagon by pancreatic α-cells plays a critical role in maintaining elevated blood glucose levels in people with T2D^14^,^24^,^25^. Researchers have been trying to develop pharmacological approaches to inhibit the release of glucagon as a new treatment option. For example, some compounds such as imidazolines^26^, somatostatin analogues^27^, amylin and pramlintide^25^,^28^ have been developed to directly inhibit glucagon secretion but have shown mixed results. Likewise, pramlintide which is an analogue of amylin had a modest effect on reducing glucagon secretion but no reported effect on reducing blood glucose in people with T2D^25^,^29^. The lack of clinical efficacy of drugs developed to reduce glucagon secretion maybe due to the complexity of the secretory pathway and the interaction with T2D itself. In particular, hyperglucagonemia in T2D is regulated by reduced SGLT2 levels in the α-cells^16^. So we would need to develop drugs that activate or increase SGLT2 levels to block glucagon secretion but this might have an impact on the kidney by increasing the reabsorption of renal glucose^16^,^30^,^31^. Therefore, a possible strategy would be to inhibit the glucagon signalling pathway.

The mechanism of action niclosamide targets leading to improved glucose metabolism in obese mice is likely via uncoupling mitochondria thus reducing the availability of cAMP needed to activate PKA signalling^18^,^20^. This is similar to the proposed mechanism of action of metformin that inhibits mitochondrial complex I which results in reduced ATP production leading to inhibition of adenylate cyclase activity and cAMP generation upon stimulation of the glucagon receptor in the liver^32^,^33^,^34^. As a result, PKA activation and its downstream pathways are inhibited.

Previously, one other study reported the efficacy of niclosamide as a potential drug for the treatment of T2D^19^. Tao *et al*.^19^ described that NENS increased energy expenditure and lipid oxidation, which dramatically improves hepatic steatosis and is efficacious in preventing and treating diabetic symptoms developed in obese mice. They also found that NENS reduced total body weight that would also impact on glucose metabolism. Interestingly, they also reported a significant reduction in hepatic glucose production during a gold standard clamp experiment^19^. This supports our findings that both NENS and Nic block the effect of glucagon in the liver that is the major driver of hepatic glucose production^17^,^35^. Although we did not find evidence of increased energy expenditure with either NENS or Nic as stated by Tao *et al*.^19^ the discrepancy in our results may be due to the duration of treatment and also differences in the timing of the drug intervention and differences in the amount of total energy derived from fat (60% vs. 40% in our study). This may also explain why we found no changes in total body composition compared to changes they found^19^. Likewise, other groups have also found no effect of Nic on total body weight that supports our findings^36^. Thus, our *in vivo* data, strongly suggest that niclosamide is reducing the effect of glucagon in the liver and improving glucose metabolism through the PKA signalling pathway.

In conclusion, this is the first study to compare the efficacy of the two forms of niclosamide (NENS or Nic) in blocking glucagon signalling in the liver to improve glucose metabolism via PKA signalling. Because we found no differences in the uncoupling properties of NENS vs. Nic and similar effects in whole animal metabolic testing, the FDA approved form of niclosamide would be an ideal drug to undertake human clinical trials in obese humans with T2D. Nic has an excellent safety profile in humans, dogs and rodents and generally accumulates in the liver thus reducing potential off target effects^19^,^37^,^38^,^39^,^40^,^41^. However, future human research studying the chronic effects of niclosamide would need to carefully study the hypoglycaemic risk^42^, hypolipidaemic^43^, and lipid synthesis^44^ effects of inhibiting glucagon signalling in the liver. In conclusion, our *in vitro*^18^ and present *in vivo* data shows a novel mechanism of action of the FDA approved anthelmintic drug niclosamide, which could provide a new and practical approach for treating T2D in people with obesity and defects in glucagon signalling.

## Methods

### Reagents

The following reagents were purchased: Insulin (Actrapid^®^) and Glucagon (Glucagen^®^Hypokit) from Novo nordisk (DK). Niclosamide ethanolamine salt; NENS (niclosamide 5-chloro-salicyl-(2-chloro-4-nitro) anilide 2-aminoethanol salt), Chemlin (Nanjing, CN). Niclosamide; Nic (2′,5-dichloro-4′-nitrosalicylanilide), Sigma-Aldrich (St. Louis, MO, USA). Glucose (D-(+) Glucose) was sourced from Sigma-Aldrich (St. Louis, MO, USA).

### Diets

The chow diet was purchased from Gordon’s Specialty Stockfeeds Pty Ltd, NSW, AU. Chow pallets were blended into a powder and drug added at 750 parts per million (p.p.m.) The diet was reconstituted with water and pellets re-modelled and dried by incubation at 46 °C for 2 hours. The total calorie content or energy density of the normal chow diet was 2.6 kcal/g and for high fat diet was 9 kcal/g. The high fat diet was made as follows: casein 522 g (Cottee, AU), sucrose 460 g (Bundaberg Suger Ltd, QLD), starch 386 g (New Zealand Starch Ltd, NZ), mineral mix 102 g (MP Biochemicals, USA), trace minerals 27.6 g (MP Biochemicals, USA), bran 114 g (Allied Mills Pyt Ltd, AU), methionine 6.8 g (Sigma-Aldrich, USA), gelatine 46 g (J. L Stewart & Son Pyt Ltd, AU), choline bitartate 9.2 g (Sigma-Aldrich, USA), safflower oil 75 ml (Proteco Gold Pyt Ltd, QLD), lard 500 g (Osha Brand, CA) and AIN-93 VX VITAMIN MIX 29.6 g (MP Biochemicals, USA). Drug was added at 1,500 p.p.m.

### NENS and Nic in uncoupling mouse hepatocytes mitochondria

C57BL/6 mice were anaesthetised (ketamine/xyalzine, 125 mg/kg and 25 mg/kg respectively) before being digested through collagenase perfusion. Liver was perfused with warm EGTA buffer (HBSS buffer, 0.5 mM EGTA) for 15 minutes, followed by perfusion with collagenase (Collagenase H, Roche, AU) in calcium buffer (HBSS buffer, 2 mM CaCl_2_). Hepatocytes were plated on collagen coated 10 cm dishes at a density of 0.5 × 10^6^ cells/ml and cultured overnight at 37 °C in M199 media (Gibco, ThermoFisher Scientific, MA, USA) supplemented with 100 nM dexamethasone and 1 nM insulin.

Whole cell respiration in primary hepatocytes was measured using a Clark electrode surrounded by a heated water jacket at 37 °C (Rank Brothers, Cambridge). Hepatocytes were trypsinised and spun down at 250 g for 3 minutes before being resuspended in warm M199 media (Gibco, ThermoFisher Scientific, MA, USA) and added to the electrode chambers. The addition of 5 μg/ml oligomycin inhibited respiration, after which 1 μM final concentration of the uncoupler NENS or Nic was administered as previously described^19^.

### Animal model and treatments

All animal experiments were approved by the UNSW Australia Animal Ethics Committee (AEC1665B) and all experiments performed in accordance with relevant guidelines and regulations at UNSW Australia. Male C57BL/6 mice (28 grams) were purchased from the Animal Resources Centre (ARC, Perth, Australia) and weight/fat matched into six groups: (1) control chow, (2) NENS chow, (3) Nic chow, (4) control high fat, (5) NENS high fat and (6) Nic high fat. Mice where fed either chow or a high fat diet and either dosed with NENS or Nic, giving an equivalent of 150 mg/kg body weight per day for each dietary group^19^. Each group was maintained on the diet or diet-drug intervention for 10 weeks.

### Body weight, food intake and body composition

Daily body weight and food intake were measured for each group. Body composition was analysed using the EchoMRI^TM^ quantitative magnetic resonance system (EchoMRI LLC, TX, USA) following manufacturer’s instructions, every 4 weeks during the study.

### Glucose tolerance test (GTT)

On the day of GTT, animals were fasted from 8.00 am to 2.00 pm and a basal blood glucose measurement taken from the tail tip. Lean mass was measured the day before experiment for each animal using EchoMRI. Mice were injected intraperitoneally (i.p.) with 20% glucose solution at a dose of 2 mg/kg total lean body mass. Blood glucose concentrations were determined using the Accu-CHEK^®^ Performa (Roche, DE) at times indicated in the figure. Blood was collected from the tail bleeds using heparinised tubes (Microvette^®^ CB 300 LH, Sarstedt, DE) at the time points 0, 15, 30 and 60 minutes and spun blood samples at 2000 g for 5 minutes at 4 °C for plasma separation. Isolated plasma was then stored at −80 °C for further analysis.

### Insulin quantification

Blood plasma samples were obtained during GTT at different time points and used for insulin levels measurement using ultra-sensitive mouse insulin ELISA kit (Crystal Chem Inc., IL, USA) followed the manufacturer’s instructions.

### Insulin sensitivity test

On the day of test, animals were fasted from 8.00 am to 2.00 pm and a basal blood glucose measurement taken. Total lean mass was measured the day before insulin sensitivity test using EchoMRI. Mice were injected with insulin i.p. at a dose of 0.75 unit/kg total lean mass based on EchoMRI. Blood glucose concentrations were measured from tail tip as indicated in the figure.

### Glucagon sensitivity test

On the day of experiment, animals were fasted from 8.00 am to 2.00 pm and a basal blood glucose measurement taken then glucagon was administered by i.p. injection at a dose of 1 mg/kg total body weight. Blood glucose concentration was measured from the tail tip 10 minutes post glucagon.

### Metabolic cage studies

Indirect energy expenditure was measured by using Columbus Laboratory Animal Monitoring System (CLAMS/Oxymax) as previously published^45^. Mice were housed individually during this procedure and maintained on their diet/drug interventions. The oxygen consumption (VO_2_), carbon dioxide production (VCO_2_), respiratory exchange ratio (RER), energy expenditure and animal movements were analysed during the experiment. All data were normalised to total lean mass using the EchoMRI. Mice were acclimatised for 24 h and data collected for 24 h.

### Tissue collection

On the day of culling, animals were in a fed state, a basal blood glucose measurement taken and glucagon was administered by i.p. injection at a dose of 1 mg/kg total body weight, after 10 minutes blood glucose concentration was measured from the tail tip. At 15 minutes post glucagon exposure, mice were euthanised by cervical dislocation and liver removed and immediately frozen in liquid nitrogen until further biochemical analysis.

### Immunoblot analysis

Liver samples were weighed and homogenised in 1:25 (wet weight/volume) for 2 × 15 s in buffer containing: 1% NP40, 10% glycerol, 137 mM NaCl, 20 mM Tris-HCl (pH 7.4), 10 mM EDTA, 1 mM EGTA (pH 8.0), 20 mM NaF, 1 mM Na_4_P_2_O_7_, 1 mM vanadate, 4 μg/ml aprotinin, 4 μg/ml leupeptin, 1 mM PMSF. After 1 h rotation at 4 °C, samples were centrifuged (11,500 *g* for 10 minutes at 4 °C) and protein content was determined (BIO-RAD, Dc Protein Assay, Hercules, CA, USA). Proteins were separated by SDS-PAGE and transferred to polyvinylidene difluoride (PVDF) membranes (Pall Corporation, Port Washington, NY, USA). Based on the molecular weight from our protein standard, membranes were cut into strips to allow multiple target analysis from the one transfer (i.e. β-catenin-92 kDa and CREB-43 kDa). The membranes were incubated for 1 h in blocking buffer (20 mM Tris; pH 7.4), 137 mM NaCl, 0.5% (v/v) Tween 20 containing 4% (w/v) BSA (ICP Bio, Auckland, New Zealand) at room temperature and then incubated at 4 °C overnight in blocking buffer containing primary β-catenin antibody (pS552 and total; 1:1000; Cell Signaling Technology, Danvers, MA, USA), CREB antibody (pS133; 1:1000; Cell Signaling Technology, Danvers, MA, USA) and GAPDH antibody (1:1000; Cell Signaling Technology, Danvers, MA, USA) which we have validated and published previously^17^,^18^,^21^. Immunoreactive proteins were detected using horseradish peroxidase-linked secondary antibody (1:1000; Cell Signaling Technology, Danvers, MA, USA) and enhanced chemiluminescence (ECL) according to the manufacturer’s instructions (BIO-RAD, Hercules, CA, USA). Image was analysed by ChemiDoc XRS (BIO-RAD, Hercules, CA, USA) and ImageJ software for quantification.

### Statistical analysis

Data are presented as the mean ± S.E.M. except where otherwise stated and statistical significance is indicated as *P < 0.05, **P < 0.01 and ***P < 0.001. Two-way ANOVA followed by Tukey’s test were performed using GraphPad Prism v6.0 software for statistical analysis and bar graphs.

## Additional Information

**How to cite this article**: Chowdhury, M. K. H. *et al*. Niclosamide reduces glucagon sensitivity via hepatic PKA inhibition in obese mice: Implications for glucose metabolism improvements in type 2 diabetes. *Sci. Rep.* **7**, 40159; doi: 10.1038/srep40159 (2017).

**Publisher's note:** Springer Nature remains neutral with regard to jurisdictional claims in published maps and institutional affiliations.

## Supplementary Material

Supplementary Information

## References

[b1] Shaw J. E., Sicree R. A. & Zimmet P. Z. Global estimates of the prevalence of diabetes for 2010 and 2030. Diabetes research and clinical practice 87, 4–14, doi: 10.1016/j.diabres.2009.10.007 (2010). 19896746

[b2] Turner N., Zeng X. Y., Osborne B., Rogers S. & Ye J. M. Repurposing Drugs to Target the Diabetes Epidemic. Trends in pharmacological sciences 37, 379–389, doi: 10.1016/j.tips.2016.01.007 (2016). 26900045

[b3] Emerging Risk Factors C. . Diabetes mellitus, fasting blood glucose concentration, and risk of vascular disease: a collaborative meta-analysis of 102 prospective studies. Lancet 375, 2215–2222, doi: 10.1016/S0140-6736(10)60484-9 (2010). 20609967 PMC2904878

[b4] Emerging Risk Factors C. . Diabetes mellitus, fasting glucose, and risk of cause-specific death. N Engl J Med 364, 829–841, doi: 10.1056/NEJMoa1008862 (2011). 21366474 PMC4109980

[b5] DeFronzo R. A. Pathogenesis of type 2 diabetes mellitus. The Medical clinics of North America 88, 787–835, ix, doi: 10.1016/j.mcna.2004.04.013 (2004). 15308380

[b6] Basu A. . Effects of a change in the pattern of insulin delivery on carbohydrate tolerance in diabetic and nondiabetic humans in the presence of differing degrees of insulin resistance. The Journal of clinical investigation 97, 2351–2361, doi: 10.1172/jci118678 (1996). 8636416 PMC507316

[b7] Butler P. C. & Rizza R. A. Contribution to postprandial hyperglycemia and effect on initial splanchnic glucose clearance of hepatic glucose cycling in glucose-intolerant or NIDDM patients. Diabetes 40, 73–81 (1991). 2015976

[b8] Larsson H. & Ahren B. Islet dysfunction in insulin resistance involves impaired insulin secretion and increased glucagon secretion in postmenopausal women with impaired glucose tolerance. Diabetes care 23, 650–657 (2000). 10834425 10.2337/diacare.23.5.650

[b9] Mighiu P. I. . Hypothalamic glucagon signaling inhibits hepatic glucose production. Nature medicine 19, 766–772, doi: 10.1038/nm.3115 (2013). 23685839

[b10] Lee Y., Wang M. Y., Du X. Q., Charron M. J. & Unger R. H. Glucagon receptor knockout prevents insulin-deficient type 1 diabetes in mice. Diabetes 60, 391–397, doi: 10.2337/db10-0426 (2011). 21270251 PMC3028337

[b11] Burcelin R., Katz E. B. & Charron M. J. Molecular and cellular aspects of the glucagon receptor: role in diabetes and metabolism. Diabetes & metabolism 22, 373–396 (1996). 8985646

[b12] Toft I., Gerich J. E. & Jenssen T. Autoregulation of endogenous glucose production during hyperglucagonemia. Metabolism: clinical and experimental 51, 1128–1134 (2002). 12200756 10.1053/meta.2002.34702

[b13] Unger R. H., Aguilar-Parada E., Muller W. A. & Eisentraut A. M. Studies of pancreatic alpha cell function in normal and diabetic subjects. J Clin Invest 49, 837–848, doi: 10.1172/JCI106297 (1970). 4986215 PMC322540

[b14] Consoli A. Role of liver in pathophysiology of NIDDM. Diabetes Care 15, 430–441 (1992). 1559410 10.2337/diacare.15.3.430

[b15] Shah P. . Lack of suppression of glucagon contributes to postprandial hyperglycemia in subjects with type 2 diabetes mellitus. The Journal of clinical endocrinology and metabolism 85, 4053–4059, doi: 10.1210/jcem.85.11.6993 (2000). 11095432

[b16] Bonner C. . Inhibition of the glucose transporter SGLT2 with dapagliflozin in pancreatic alpha cells triggers glucagon secretion. Nat Med 21, 512–517, doi: 10.1038/nm.3828 (2015). 25894829

[b17] Chowdhury M. K. . Glucagon phosphorylates serine 552 of beta-catenin leading to increased expression of cyclin D1 and c-Myc in the isolated rat liver. Archives of physiology and biochemistry 121, 88–96, doi: 10.3109/13813455.2015.1048693 (2015). 26135564

[b18] Chowdhury M. K. . Niclosamide blocks glucagon phosphorylation of Ser552 on beta-catenin in primary rat hepatocytes via PKA signalling. The Biochemical journal 473, 1247–1255, doi: 10.1042/bcj20160121 (2016). 26964897

[b19] Tao H., Zhang Y., Zeng X., Shulman G. I. & Jin S. Niclosamide ethanolamine-induced mild mitochondrial uncoupling improves diabetic symptoms in mice. Nat Med 20, 1263–1269, doi: 10.1038/nm.3699 (2014). 25282357 PMC4299950

[b20] Delghandi M. P., Johannessen M. & Moens U. The cAMP signalling pathway activates CREB through PKA, p38 and MSK1 in NIH 3T3 cells. Cellular signalling 17, 1343–1351 (2005). 16125054 10.1016/j.cellsig.2005.02.003

[b21] Emmanuelle C., Coralie G. D., Deborah L. H. & Peter R. S. Identification of a pathway by which glucose regulates beta-catenin signalling via the cAMP/protein kinase A pathway in beta-cell models. Biochemical Journal 449, 803–811 (2013). 23198873 10.1042/BJ20121454

[b22] Mayr B. & Montminy M. Transcriptional regulation by the phosphorylation-dependent factor CREB. Nature reviews Molecular cell biology 2, 599–609 (2001). 11483993 10.1038/35085068

[b23] Jiang G. & Zhang B. B. Glucagon and regulation of glucose metabolism. American Journal of Physiology-Endocrinology And Metabolism 284, E671–E678 (2003). 12626323 10.1152/ajpendo.00492.2002

[b24] Kahn S. E., Cooper M. E. & Del Prato S. Pathophysiology and treatment of type 2 diabetes: perspectives on the past, present, and future. Lancet 383, 1068–1083, doi: 10.1016/S0140-6736(13)62154-6 (2014). 24315620 PMC4226760

[b25] Quesada I., Tudurí E., Ripoll C. & Nadal Á. Physiology of the pancreatic α-cell and glucagon secretion: role in glucose homeostasis and diabetes. Journal of Endocrinology 199, 5–19 (2008). 18669612 10.1677/JOE-08-0290

[b26] Zaitsev S. V. . Imidazoline compounds stimulate insulin release by inhibition of KATP channels and interaction with the exocytotic machinery. Diabetes 45, 1610–1618 (1996). 8866568 10.2337/diab.45.11.1610

[b27] Strowski M. Z. . Antidiabetic activity of a highly potent and selective nonpeptide somatostatin receptor subtype-2 agonist. Endocrinology 147, 4664–4673, doi: 10.1210/en.2006-0274 (2006). 16857751

[b28] Dunning B. E., Foley J. E. & Ahren B. Alpha cell function in health and disease: influence of glucagon-like peptide-1. Diabetologia 48, 1700–1713, doi: 10.1007/s00125-005-1878-0 (2005). 16132964

[b29] Young A. Inhibition of glucagon secretion. Advances in pharmacology (San Diego, Calif.) 52, 151–171, doi: 10.1016/s1054-3589(05)52008-8 (2005). 16492545

[b30] Mudaliar S., Polidori D., Zambrowicz B. & Henry R. R. Sodium-Glucose Cotransporter Inhibitors: Effects on Renal and Intestinal Glucose Transport: From Bench to Bedside. Diabetes care 38, 2344–2353, doi: 10.2337/dc15-0642 (2015). 26604280

[b31] Wright E. M., Loo D. D. & Hirayama B. A. Biology of human sodium glucose transporters. Physiological reviews 91, 733–794, doi: 10.1152/physrev.00055.2009 (2011). 21527736

[b32] Detaille D., Guigas B., Leverve X., Wiernsperger N. & Devos P. Obligatory role of membrane events in the regulatory effect of metformin on the respiratory chain function. Biochemical pharmacology 63, 1259–1272 (2002). 11960602 10.1016/s0006-2952(02)00858-4

[b33] Owen M. R., Doran E. & Halestrap A. P. Evidence that metformin exerts its anti-diabetic effects through inhibition of complex 1 of the mitochondrial respiratory chain. The Biochemical journal 348(Pt 3), 607–614 (2000). 10839993 PMC1221104

[b34] Zhou G. . Role of AMP-activated protein kinase in mechanism of metformin action. The Journal of clinical investigation 108, 1167–1174, doi: 10.1172/jci13505 (2001). 11602624 PMC209533

[b35] Ramnanan C. J., Edgerton D. S., Kraft G. & Cherrington A. D. Physiologic action of glucagon on liver glucose metabolism. Diabetes, obesity & metabolism 13 Suppl 1, 118–125, doi: 10.1111/j.1463-1326.2011.01454.x (2011). PMC5371022 21824265

[b36] Ye T. . The anthelmintic drug niclosamide induces apoptosis, impairs metastasis and reduces immunosuppressive cells in breast cancer model. PloS one 9, e85887, doi: 10.1371/journal.pone.0085887 (2014). 24416452 PMC3885752

[b37] Chen M. . The anti-helminthic niclosamide inhibits Wnt/Frizzled1 signaling. Biochemistry 48, 10267–10274, doi: 10.1021/bi9009677 (2009). 19772353 PMC2801776

[b38] Gemmell M. A., Johnstone P. D. & Oudemans G. The effect of niclosamide on Echinococcus granulosus, Taenia hydatigena and Taenia ovis infections in dogs. Research in veterinary science 22, 389–391 (1977). 877439

[b39] Pearson R. D. & Hewlett E. L. Niclosamide therapy for tapeworm infections. Annals of internal medicine 102, 550–551 (1985). 3977200 10.7326/0003-4819-102-4-550

[b40] Andrews P., Thyssen J. & Lorke D. The biology and toxicology of molluscicides, Bayluscide. Pharmacology & therapeutics 19, 245–295 (1982). 6763710 10.1016/0163-7258(82)90064-x

[b41] Hecht G. & Gloxhuber C. [Studies on the tolerance of 5,2′-dichloro-4′-nitrosalicylanilide ethanolamine salt]. Zeitschrift fur Tropenmedizin und Parasitologie 13, 1–8 (1962). 13905830

[b42] Ali S. & Drucker D. J. Benefits and limitations of reducing glucagon action for the treatment of type 2 diabetes. American journal of physiology. Endocrinology and metabolism 296, E415–421, doi: 10.1152/ajpendo.90887.2008 (2009). 19116373

[b43] Guettet C. . Effect of chronic glucagon administration on lipoprotein composition in normally fed, fasted and cholesterol-fed rats. Lipids 26, 451–458 (1991). 1881241 10.1007/BF02536072

[b44] Longuet C. . The glucagon receptor is required for the adaptive metabolic response to fasting. Cell metabolism 8, 359–371, doi: 10.1016/j.cmet.2008.09.008 (2008). 19046568 PMC2593715

[b45] Smith G. C. . Effects of acutely inhibiting PI3K isoforms and mTOR on regulation of glucose metabolism *in vivo*. The Biochemical journal 442, 161–169, doi: 10.1042/bj20111913 (2012). 22142257 PMC3343648

