# Supplementary material for: Niclosamide reduces glucagon sensitivity via hepatic PKA inhibition in obese mice: Implications for glucose metabolism improvements in type 2 diabetes
Source: Sci Rep. 2017 Jan 5;7:40159. doi: 10.1038/srep40159 (PMC5214666; doi:10.1038/srep40159)
Supplement: Supplementary Information [file srep40159-s1.pdf]

Supplementary Information

Niclosamide reduces glucagon sensitivity via hepatic PKA inhibition in obese mice: Implications for glucose metabolism improvements in type 2 diabetes.

Md. Kamrul Hasan Chowdhury[1], Nigel Turner[1], Nicholas L Bentley[1], Abhirup Das[1], Lindsay E Wu[1], Dulama Richani[2], Sonia Bustamante[3], Robert Gilchrist[2], Margaret J Morris[1] Peter R Shepherd[4] and Greg C Smith[1,*]

[1]Depatment of Pharmacology, School of Medical Sciences, UNSW Australia, Sydney, Australia

[2]School of Women's & Children Health, UNSW Australia, Sydney, Australia

[3]Bioanalytical Mass Spectrometry Facility, UNSW Australia, Sydney, Australia

[4]Department of Molecular Medicine & Pathology, University of Auckland, Auckland, New Zealand

Correspondence

Greg C. Smith

Telephone: +61 (2) 93858075

Fax: +61 (2) 93850023

E-mail: g.smith@unsw.edu.au

Supplementary Figure S1

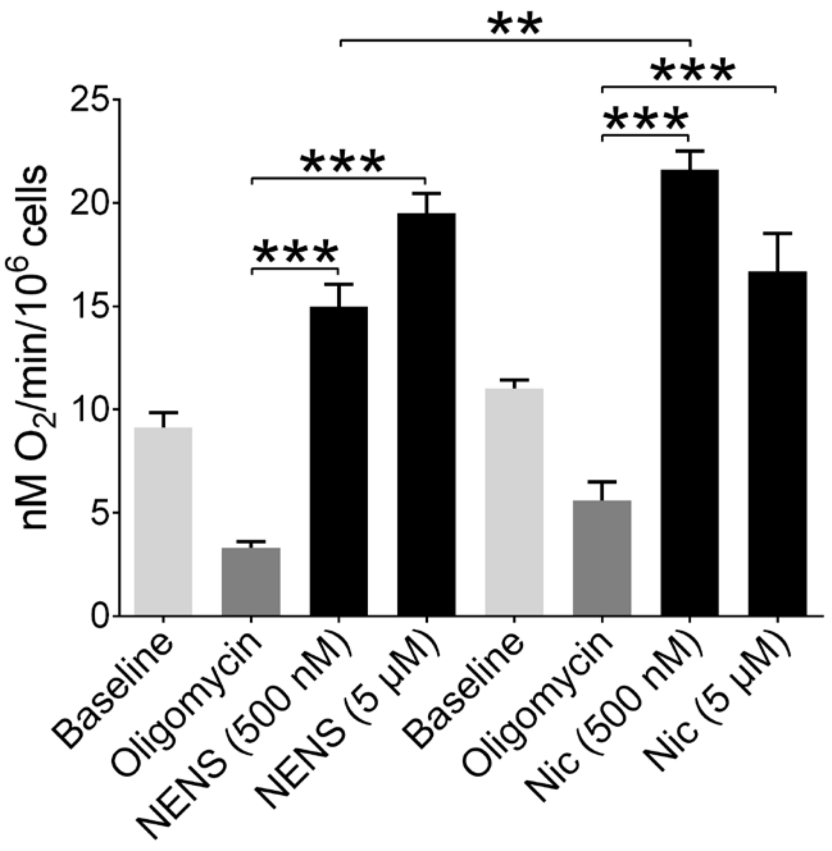

Supplementary Figure S1. NENS or Nic uncouples mitochondrial respiration. Oxygen consumption of isolated C57BL/6 hepatocytes using the Clark electrode with oligomycin and two forms of niclosamide (NENS or Nic; 500 nM and 5 µM). Each experiment is in duplicate and repeated three times independently. Statistical significance (P) was determined by multiple comparisons Two-way ANOVA test. The data is showed as the mean ± S.E.M. *p<0.01 or ***p<0.001.
